# Mechanical and Air Permeability Performance of Novel Biobased Materials from Fungal Hyphae and Cellulose Fibers

**DOI:** 10.3390/ma14010136

**Published:** 2020-12-30

**Authors:** Inese Filipova, Ilze Irbe, Martins Spade, Marite Skute, Inga Dāboliņa, Ilze Baltiņa, Linda Vecbiskena

**Affiliations:** 1 Latvian State Institute of Wood Chemistry, Dzerbenes 27, LV-1006 Riga, Latvia; ilzeirbe@edi.lv (I.I.); martins.spade@gmail.com (M.S.); polarlapsa@inbox.lv (M.S.); linda.vecbiskena@gmail.com (L.V.); 2 Research Laboratory of Ergonomics Electrical Technologies, Institute of Industrial Electronics and Electrical Engineering, Faculty of Electrical and Environmental Engineering, Riga Technical University, Paula Valdena 1-102, LV-1048 Riga, Latvia; Inga.Dabolina@rtu.lv; 3 Institute of Design Technologies, Faculty of Materials Science and Applied Chemistry, Riga Technical University, Kipsalas 6-222, LV-1048 Riga, Latvia; Ilze.Baltina@rtu.lv

**Keywords:** fungal fibers, cellulose fibers, microstructure, mechanical properties, air permeability, biodegradation, mushroom paper

## Abstract

Novel biobased materials from fungal hyphae and cellulose fibers have been proposed to address the increasing demand for natural materials in personal protective equipment (PPE). Materials containing commercially available kraft fibers (KF), laboratory-made highly fibrillated hemp fibers (HF) and fungal fibers (FF) obtained from fruiting bodies of lignicolous basidiomycetes growing in nature were prepared using paper production techniques and evaluated for their mechanical and air permeability properties. SEM and microscopy revealed the network structure of materials. The tensile index of materials was in the range of 8–60 Nm/g and air permeability ranged from 32–23,990 mL/min, depending on the composition of materials. HF was the key component for strength; however, the addition of FF to compositions resulted in higher air permeability. Chemical composition analysis (Fourier-transform infrared spectroscopy) revealed the presence of natural polysaccharides, mainly cellulose and chitin, as well as the appropriate elemental distribution of components C, H and N. Biodegradation potential was proven by a 30-day-long composting in substrate, which resulted in an 8–62% drop in the C/N ratio. Conclusions were drawn about the appropriateness of fungal hyphae for use in papermaking-like technologies together with cellulose fibers. Developed materials can be considered as an alternative to synthetic melt and spun-blown materials for PPE.

## 1. Introduction

The use of bio-materials is driven not only by European regulations, but also by people’s desire to use natural materials and to live in a cleaner environment. A significant part of the scientific force is devoted to the investigation of renewable and sustainable biobased materials and their possible applications [1]. The global challenge is to make most of the short-service-life items biodegradable to avoid creating long-term pollution. Despite the progress towards replacing plastics with bio-materials, several related challenges are still present, and even expanding [2]. The current pandemic caused demand for disposable face cover items mostly made of synthetic polymers, such as polypropylene, polyethylene, polyesters, polyamines, polycarbonates and polyphenylene oxide [3]. These materials are not biodegradable and can cause increased pollution to the environment due to improper waste management [4]; therefore, they have to be replaced with more sustainable materials. Intensive work is being done all over the world on research on biodegradable polymers applicable for personal protective equipment (PPE) [5]. However, there are natural polymers and even micro and macro fibers in nature with insufficiently explored potential for use in the creation of different materials and for different applications, such as biodegradable filtering materials for PPE. A medical face mask is a medical device (also used as PPE and/or collective protective equipment) that covers the mouth and nose, providing a barrier to reduce the direct spread of infectious microorganisms between medical staff and patients. Medical face masks can also be worn by patients and others to reduce the risk of spreading the infection, especially in the event of an epidemic or pandemic. Medical face masks usually consist of a layer of filter placed, sewn, glued, or pressed between outer layers.

Cellulose fibers from non-wood plants are the one of the most suitable sources for sustainable materials, not only because of world abundance, but also because of their short growing period, which is the most significant advantage compared with wood cellulose. Hemp fibers are among the most important industrial sources of cellulosic fiber and are considered as one of the strongest members of bast natural fibers family, which is derived from the hemp plant under the species of Cannabis. Hemp fibers are the main constituent of the hemp plant outer stem and have been used for cordage, pulp, textile and recycling additive purposes [6], as thermal and acoustic insulation material [7] and for reinforcing of composite materials. These fibers are known for their biodegradability and low density compared with artificial fibers, as well as for their inherent mechanical, thermal, and acoustic properties [8,9]. They are often used in bioethanol production [10]. The length of hemp fiber can be up to 5 cm, while wood pulp fibers are generally 0.7–2.5 mm long.

Along with other bio-based materials, mycelium and lignocellulosic composite-based materials have recently been attracting scientific and practical attention [11]. Bioactive ultrafiltration membranes [12] and highly porous carbon-fiber monolith from filamentous fungi have been created [13]. Fungus hyphae is chitinous microfiber that grows fast—taking only several days to grow from single cells to a macro scale—and has a length of centimeters and a diameter less than 10 μm. Due to the large quantities of function groups on the cell wall of hyphae, such as phosphonate, hydroxyl and amine groups, fungus fibers are promising for new functional bio-based materials [14,15,16]. Although handmade so-called mushroom papers have been known for decades and many appropriate fungus species (e.g., *Trametes versicolor*, *Ganoderma* and *Fomitopsis* species) and methodologies are available [17,18], non-wowen or paper-like materials from released fungus hyphae and cellulose fibers have not been investigated in detail or appeared in published research.

The aim of current research was to prepare biodegradable composite materials from the pulp of basidiomycete *Ganoderma applanatum* together with cellulose fibers obtained both from wood and hemp and investigate their mechanical and air permeability properties with regard to their potential use as a replacement for the synthetic materials used in PPE.

## 2. Materials and Methods

Bleached softwood kraft fibers (KF) were provided by Metsä Fibre (Espoo, Finland) as pressed sheets and used without specific pretreatment.

Fungal hyphae were obtained from naturally growing fruiting bodies of basidiomycete *Ganoderma applanatum*. The fruiting bodies were dried at room temperature and cut in smaller pieces (3 cm × 3 cm). Sample pieces were kept in 4% NaOH solution for 24 h at room temperature in order to remove proteins and alkali soluble polysaccharides, as well as to soften and swell the fungal biomass. Following this, NaOH extraction samples were washed in tap water and mechanically disintegrated using Blendtec 725 (Orem, UT, USA) at 360 W for 30 s. Obtained fungal hyphae pulp (FF) was dried at room temperature and kept in a dry state until used.

Hemp fibers (HF) were obtained from industrial hemp *Cannabis sativa* (USO-31). The decortication fibers were treated in 4% NaOH solution at 165 °C for 75 min, then washed in tap water to neutralize. They were then refined using Blendtec 725 (Orem, UT, USA) at 179 W for 7 min at 1.5% consistency, dried at room temperature and kept in a dry state until used.

Dimensions of fibers were determined with a FiberTester (Lorentzen & Wettre, Stockholm, Sweden) and drainability was determined with a Schopper-Riegler Freeness Tester according to ISO 5267-1:1999.

For composite production, a certain amount (in grams) of fiber pulp was placed in a glass baker and soaked in 1–2 L of distilled water for 8 h, then disintegrated using 75,000 revolutions in the disintegrator (Frank PTI, Laakirchen, Austria). Material sheets were produced according to ISO 5269-2:2004 with a Rapid Köthen paper machine (Frank PTI, Laakirchen, Austria). Samples were named according to their composition and included the abbreviations KF, HF and FF. The components shown in the composites were in equal mass proportions; for example, KF HF FF meant the mass of each component was one third of the total mass, and KF FF indicated that each component was half of the total mass. Each composition was prepared at four different grammages (20–80 g/m^2^) and at least five parallel samples were investigated for each case.

Thickness of the material was measured according to ISO 534:2011 with a micrometer F16502 (Frank-PTI, Laakirchen, Austria). Grammage (weight per unit area in g/m^2^) of samples was calculated by dividing the mass with area according to ISO 536:2019.

The microstructure was examined by a light microscope Leica DMLB at a magnification of 200×. The images were captured using a video camera Leica DFC490 using calibrated image analysis software Image-Pro plus 6.3 (Media Cybernetics, Inc., Rockville, MD, USA).

For scanning electron microscopy (SEM) images, samples (surface; cross sections of cut and torn samples) were coated with gold plasma using a K550X sputter coater (Emitech, Ashford, UK) and examined with Vega TC (Tescan, Brno-Kohoutovice, Czech Republic) with software 2.9.9.21.

Air permeability was tested according to EN 14683:2019 and ISO 9237:1995 using an air permeability tester M021S (SDL Atlas, Rock Hill, SC, USA) with a head test area of 5 cm^2^, and according to ISO 5636-3:2013 using an air permeability tester 266 (Lorentzen & Wettre, Stockholm, Sweden). Eight different commercially available disposable face masks consisting of three layers made of polypropylene spunbond-meltblown-spunbond nonwoven fabrics were tested for air permeability as comparison for developed samples.

Mechanical testing samples with a width of 1 cm were prepared with a strip cutter (Frank-PTI, Laakirchen, Austria) and tested according to ISO 1924-1:1992 and ISO 2758:2014 using a tensile tester vertical F81838 and burst tester (both from: Frank-PTI, Laakirchen, Austria).

FTIR spectrum values were recorded using a Nicolet iS50 spectrometer (Thermo Fisher Scientific, Waltham, MA, USA) at a resolution of 4 cm^−1^ and recording 32 scans per sample. The total content of carbon, hydrogen and nitrogen was determined with VarioMACRO (Elementar Analysensysteme, Langenselbold, Germany).

A general test for predicting the potential of biodegradability of the samples was carried out using an OxiTop OC 110 respirometric system (WtW, Weilheim, Germany) and performing a 30 day composting test on 100 g of the compost substrate (SIA Zeltābele, Saldus, Latvia) with moisture at 78%. Contents composed of the following were placed in a bottle: N ≤ 0.4%, P_2_O_5_ ≤ 0.15%, K_2_O ≤ 0.06%, organics ≤ 16% and a pH 6–7; 0.5 g of composite sample was added. The incubation was performed at 20 °C without agitation.

Statistical analysis of obtained results was performed using an Excel 2016 MSO data statistical analysis tool.

## 3. Results and Discussion

### 3.1. Fiber Properties

The fiber length, width, shape factor or straightness, and freeness degree of KF, HF and FF are given in Table 1. The size of ordinary softwood kraft fiber corresponds to the generally accepted numbers [19] as this fiber is one of the mostly used fiber types for papermaking. Hemp fibers were 1.28 mm long and 21.3 µm wide, demonstrating a size more similar to hardwood fiber [20]. The size and properties of cellulose fibers usually depend on the isolation method and parameters of pre- or post- treatment [21]. It was clearly demonstrated, via the freeness number of 91.5 °SR for HF, that the properties of the cellulose fiber were affected by mechanical treatment after production. Milling or refining is a method used to increase the ability of fiber to bond via defibrillation, thus increasing the surface area fiber and mechanical strength of the final material [21]. The fungus fiber or fungal hyphae showed a significantly different size compared to cellulose fibers. The width of FF measured using microscopy and SEM was 3–6 µm; however, the length was not possible to measure precisely, because FF appeared as aggregates of hyphae not as individual units. The mechanical force applied to the fungal biomass during the disintegration had a significant effect on the restructuration of the hyphae network, and the fruiting body was transformed to pulp; however, the process did not release totally individual fibers. Nevertheless, it was clearly shown in SEM images that visible and identifiable parts or fragments of individual hyphae were more than 100–300 µm long. To our knowledge, the freeness of hyphae has previously appeared in published research; therefore, the obtained number of 21.5 °SR is interesting from an engineering and material production perspective, because it demonstrates the drainage ability; this is important in such technologies as papermaking, where water is being added and drained during the process. The freeness number of FF corresponds to non-refined chemo-mechanical wood pulp [22] and allows for the assessment of FF as appropriate for materials produced by papermaking-like technologies.

### 3.2. Structure of Materials

**Figure 1:**
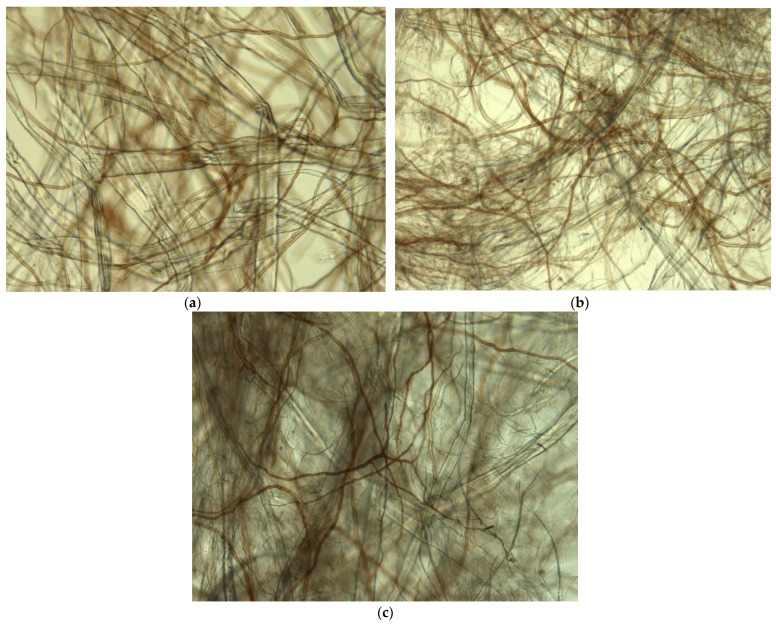
Microstructure (light microscopy, magnification 200×) of the composite materials: (**a**) KF FF; (**b**) HF FF; (**c**) HF KF FF.

The microstructure of the fungal hyphae containing materials KF FF, HF FF and HF KF FF is shown in Figure 1. The composition of kraft fibers, hemp fibers and fungal fibers is mechanically bound together, making a net of composite material with different mechanical and air permeability properties. The structure of materials is affected by the individual properties of fibers (Table 1). The fungal fibers in the form of bundles are randomly distributed within the net cellulose fibers.

**Figure 2:**
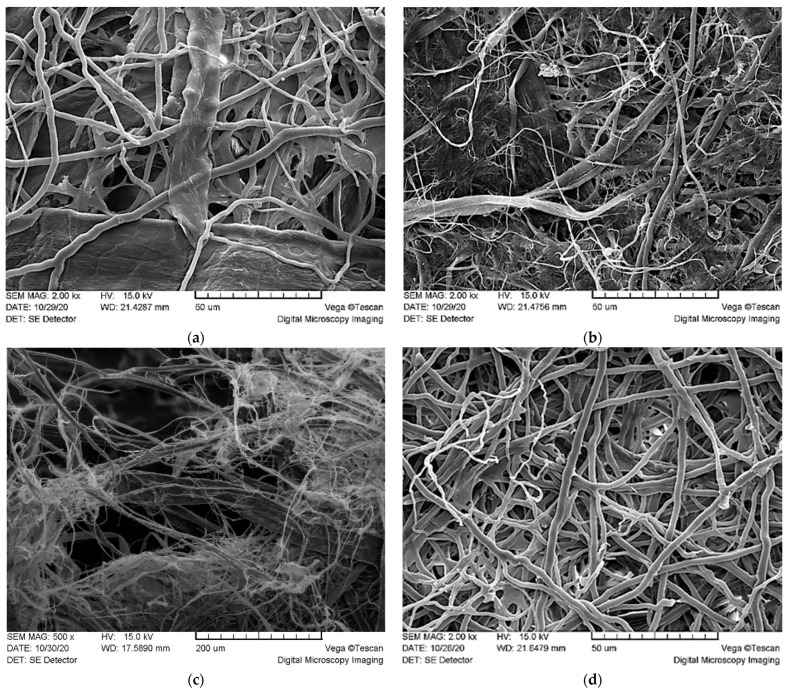
Ultrastructure (SEM, magnifications are given after sample name) of the materials: (**a**) KF FF, 1000×; (**b**) HF FF, 2000×; (**c**) HF KF FF, 500×; (**d**) FF, 2000×.

SEM images (Figure 2) reveal the detailed ultrastructure of composite materials. Images confirm the observations of light microscopy. Fiber orientation appears to be randomly distributed; entangled fibers are mechanically bound together with H bonds, forming a non-oriented, multi-layered network. The KF FF surface structure shows round fungal fibers (3–6 µm) (skeletal hyphae) within the flat-shaped kraft cellulose fibers (30 µm) (Figure 2a). The structure of the HF FF material consists of round fungal fibers and hemp fibers with variable diameters (Figure 2b). The flattened hemp fibers having a diameter of 10–20 µm are incorporated in a dense network of microfibers of approximately 3 µm in diameter. The fractured surface of HF KF FF material shows a typical multi-layered, rather loose network with pores (Figure 2c). The orientation of fibers in composite materials is irregular. Thin fungal fibers and hemp microfibers decrease the porosity of material, which could affect air permeability. The FF material, consisting only of fungal hyphae (Figure 2d) shows a rather uniform structure of nonwoven material, which can be at least visually compared to melt blown webs of polyethylene fibers used for filtering layers of medical personal protective equipment, where fiber entanglement and bonding at a high degree is a requirement [23]. The detected diameter of hyphae used in this research is smaller than the diameter of melt blown polymer fiber in nonwovens, which is in the range of 11.5 µm to 24.7 µm, depending on the production parameters [24].

### 3.3. Air Permeability

The air permeability results of materials according to different standards are shown in Table 2. Materials containing hemp fibers showed the lowest air permeability values. Microfibrils of hemp cellulose shown in SEM pictures (Figure 2c,d) formed dense networks with smaller pores because of the high surface area. The HF added to the composition significantly decreased the air permeability, which is a marginal property for materials used in face masks.

Adding an equal amount of fungal hyphae pulp to the cellulose fibers increased the air permeability for the material. The value increased almost three times (from 8275 mL/min to 23,990 mL/min) when comparing KF with KF FF and more than twice (from 32 mL/min to 77 mL/min) when comparing HF and HF FF, demonstrating the ability of fungal hyphae to improve the air permeability of cellulose fiber materials.

The air permeability of polyethylene melt blown nonwovens usually used for the production of disposable face masks is 40–200 cm^3^/s/cm^2^ according to ASTM D737 [23], which is significantly higher than the properties of investigated materials, when recalculated to the units of measurements used according the standards EN 14,683 and ISO 9237. At the same time, the grammage or basis weight (g/m^2^) of nonwoven melt blown polymer materials used for the filtering layers of disposable face masks is up to 40 g/m^2^ [24], which is slightly lower than the values of materials used in the research. Commercially available face masks also demonstrated higher air permeability than developed materials. Overall, air permeability values of fungal hyphae containing materials do not reach the breathability numbers required for personal protection equipment. However, the research showed good correlation (correlation coefficient in the range of 0.5–0.9) between air permeability values and the grammage of materials; therefore, it is possible to increase the air permeability via decreasing the grammage during production by changing the amount of fibers used per area unit. The most promising material for air permeability is KF FF, which consists of kraft cellulose fibers and fungal hyphae.

Materials used for PPE must be assessed for their air permeability properties not only in dry state, but also considering humidity, which is present both in the air and in the human breath. Cellulose in its native form is known as hydrophilic and hygroscopic material. The impact of humidity on air permeability was not evaluated at this development stage of hyphae-cellulose material; however, it will be done in future, and the results of mechanical testing predict the strong influence of the FF amount in composition on the behavior of material, because properties of chitin differ from those of cellulose regarding moisture absorption.

### 3.4. Mechanical Properties

The mechanical properties of material are important for the assessment of its potential for appropriate applications. Tensile index (TI, Nm/g), burst index (BI, kPa m^2^/g), tensile energy absorption (TEA, J/m), breaking length (BL, km) and stretch (S, %) were evaluated and compared for all obtained materials (Table 3).

In order to compare the mechanical properties of different compositions, samples with an average grammage of 55 g/m^2^ were chosen.

The statistical analysis revealed evidence of the significant (*p* < 0.01) differences in all the results of mechanical properties among the different compositions.

Diverse correlation between mechanical properties and grammage of the samples for different compositions were detected. In the case of HF FF, a rather high (>0.98) correlation between grammage and all mechanical properties was observed; FF showed good correlation for BI (>0.99) and TEA (>0.81); both KF FF and KF HF FF showed good correlation for TEA (>0.96 and >0.99, respectively) and S (>0.81 and >0.99, respectively). Other samples did not show significant correlation, thus indicating that grammage is not the most significant factor for the mechanical performance of materials containing fungus hyphae.

**Figure 3:**
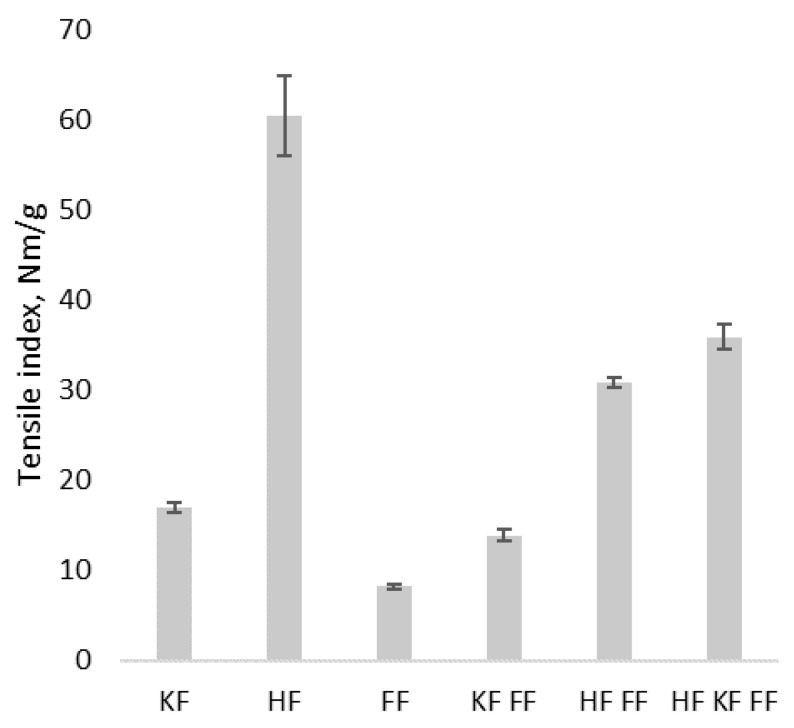
Tensile index of composite materials in dry state. KF––kraft fibers, HF––hemp fibers, and FF––fungus fibers.

Materials containing hemp fibers, HF, HF FF and KF HF FF, had the highest mechanical strength (Figure 3).

The tensile index of FF and KF FF increased by adding a certain amount of hemp fibers to the composition. The tensile strength of HF FF (30.8 Nm/g) was more than three times higher than of FF (8.2 Nm/g) and the tensile strength value of KF HF FF (35.9 Nm/g) was more than twice as high as that of KF FF (13.9 Nm/g). Results showed the highly fibrillated hemp fiber as the component responsible for strength in investigated materials. It was also concluded that materials containing only fungal hyphae had the lowest strength properties among investigated materials; however, values were still comparable. If we compare FF, for instance, with hygienic paper, the value of the tensile index, 8.2 Nm/g, was higher than the same parameter for low grammage hygienic papers [25]. Results for the tensile index in wet state were significantly lower than those in dry state because of the hydrophilicity of materials and due to the water presence causing weakening of hydrogen bonding network of cellulose fibers. However, results showed the impact of FF on the mechanical strength of material. The tensile index in wet state was more than 60% higher for KF FF than for KF.

The mechanical properties of investigated materials strongly depended on composition. Highly fibrillated HF had the most significant impact on the mechanical strength of material.

The tensile strength of melt blown polymer materials is in the range of 0.3 to 3 N and depends on the production process parameters [24]. However, the standards for disposable face masks and respirators do not include mechanical performance requirements for the equipment itself nor for filter material layers [26]. This allows us to conclude that the mechanical strength of the filter layer is not important unless it is possible to handle and process it in order to integrate it into personal protection equipment.

### 3.5. Chemical Properties

#### 3.5.1. FTIR Results

**Figure 4:**
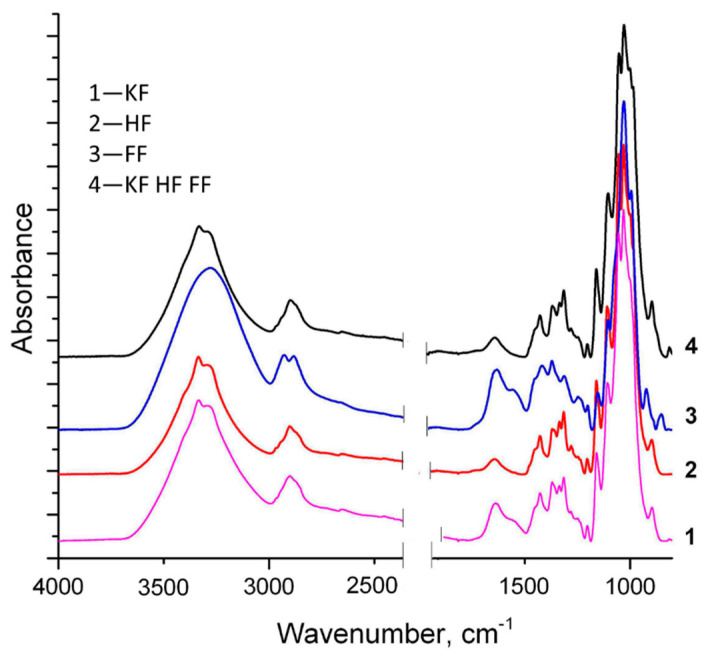
FTIR spectra of bleached softwood kraft fibers (KF), hemp fibers (HF), fungus fibers (FF) and material made of fiber composition (KF HF FF).

In order to analyze the chemical content of materials, the FTIR spectra of each mono-fiber material was first investigated and then compared to mixed fiber material KF HF FF (Figure 4). Distinct and also matching absorbance results have been observed in the results for fibers, which is caused by the polysaccharide nature and structure of both main chemical compounds involved—cellulose and chitin. In the case of FF the broad band at 3400 cm^−1^ was attributed to the –NH_2_ and –OH groups’ stretching vibration and intermolecular hydrogen bonding [27]. The bands at 2929 cm^−1^ and 2880 cm^−1^ represented the C–H symmetric stretching, and have been reported as representative bands for chitin [28]. Bands at 1640 cm^−1^ (C–O stretching) and 1550 cm^−1^ (N–H bending) were related to the amide I and amide II, respectively. Region 1160–980 cm^−1^ is typical for saccharides of fungus biomass, and C–C and C–O stretching vibrations in glycosidic bonds and pyranoid rings have been detected [29]. In the spectra of KF and HF, typical bands of cellulose are shown. The broad band observed at ~3400 cm^−1^ is attributed to O–H vibration, mainly caused by the hydrogen bonds in the cellulose [30], the band at ~2900 cm^−1^ is associated with C–H stretching vibrations, and the peak at ~1640 cm^−1^ is related to the absorbed water in carbohydrates. The peak at 1430 cm^−1^ corresponds to CH_2_ bending vibration in the cellulose [31], and sharp peaks at 1055 cm^−1^ and 897 cm^−1^ are associated with the C–O stretching and C1–H deformation vibrations of cellulose [32]. The spectra of material consisting of three fiber types, KF HF FF, had all the typical cellulose and chitin absorbance peaks and bands; no other new peaks appeared. Therefore, it is clear that only mechanical and hydrogen bonding exists among fibers in materials consisting of any composition of three initial fibers.

#### 3.5.2. Elemental Composition Analysis

The presence of N-containing components in fungus-containing materials is confirmed also by elemental compound analysis (Table 4). There is more N in materials containing fungal hyphae. In the FF material, the N content is 1.8%; in the KF FF, it is 0.9%; in KF HF FF, it is 0.7%. The N content correlated with the FF amount ratio in the composition of material. Individual elements are associated with fungal cellular functions. C is a structural element of fungal cells in combination with hydrogen, oxygen, and nitrogen. N is structurally and functionally linked with cellular functions as organic amino nitrogen in proteins and enzymes. H is linked with the transmembrane proton motive force vital for fungal nutrition [33].

Elemental analysis results clearly showed the carbohydrate nature of fibers used for tested materials. Polysaccharides found in the Kraft pulp and hemp were cellulose and hemicelluloses [34].

### 3.6. Biodegradation

The biodegradation potential assessment was made using a composting test; this allowed for the evaluation of the amount of oxygen required for microbial metabolism and C/N ratio changes.

The C/N ratio represents the index of maturity for organic substances, as it significantly affects the microbiological growth. The concentration of C and N in composted samples after 30 days is shown in Table 4. Significant differences among the tested samples were found. The C/N ratio for the samples varied from 17–175.

The decrease of the C/N ratio by 8–62% of hyphae and cellulose materials, showed efficient conversion of organic matter in comparison with the raw materials. The higher the number, the higher the efficiency. Therefore, KF HF FF showed the highest decrease from composites and the most effective conversion. One of the explanations also usable for HF is that hemp cellulose fibers had a high freeness degree; therefore, high fibrillation levels resulted in increased surface area accessible for microorganisms, which are responsible for biodegradation.

Cellulose-containing substrates are the main source of energy to drive the biological transformations and chemical changes that are associated with composting. Because the reaction proceeds through biochemical pathways, proteins and enzymes are essential, and there has to be a sufficient amount of nitrogen present in the mixture relative to the amount of carbon [35].

## 4. Conclusions

Fungal hyphae or fibers (FF) is an interesting raw material with a microfiber width of 3–6 µm and a length of more than 100–300 µm. FF is appropriate for materials produced by papermaking-like technologies because of the size, freeness degree and properties of obtained materials. Biodegradable composite materials from the pulp of basidiomycete *Ganoderma applanatum*, together with cellulose fibers obtained both from wood (KF) and hemp (HF), were produced and tested with a focus on the replacement of synthetic materials in application for personal protection equipment. Microscopy and SEM revealed randomly distributed entangled fibers, forming a non-oriented, multi-layered network. Adding equal amounts of FF to the cellulose fibers increased the air permeability of material and decreased the mechanical strength in dry state; however, it increased the tensile strength in wet state. Air permeability values of tested fungal hyphae containing materials do not reach the breathability numbers required for personal protection equipment; nonetheless, it is possible to increase the air permeability via decreasing the grammage. Chitin and cellulose were detected by FTIR, and elemental analysis confirmed the presence of nitrogen. The biodegradation potential of novel materials was proven by a 30 day composting in substrate, which resulted in an 8–62% drop in the C/N ratio. Developed materials can be considered as an alternative to synthetic melt and spun blown materials for PPE.

## Figures and Tables

**Table 1:** Properties of cellulose and fungal fibers used for composite materials.

Property	KF Kraft Fibers	HF Hemp Fibers	FF Fungus Fibers
Fiber length, mm	2.1	1.28	>0.1
Fiber width, µm	29.7	21.3	3–6
Shape factor, %	82.2	82.7	-
Freeness, °SR	11	91.5	21.5

**Table 2:** Air permeability of composite materials and disposable face masks (± standard deviation). KF––kraft fibers, HF––hemp fibers, and FF––fungus fibers.

Sample	ISO 5636-3 mL/min	EN 14683 Pa/cm^2^	ISO 9237 mm/s
KF	8275 ± 491	355 ± 21	17 ± 1
HF	32 ± 2	4.3 × 10^5^ ± 2.5 × 10^3^	<1
FF	6935 ± 323	445 ± 26	13 ± 1
KF FF	23990 ± 965	91 ± 5	65 ± 4
HF FF	77 ± 2	1.4 × 10^5^ ± 8.4 × 10^3^	<1
KF HF FF	115 ± 10	8.5 × 10^4^ ± 5 × 10^2^	<1
Disposable face masks	65,054 ± 13,324	26 ± 7	241 ± 63

**Table 3:** Mechanical properties of materials. KF––kraft fibers, HF––hemp fibers, and FF––fungus fibers.

**Sample**	Tensile Index, Dry Nm/g	Tensile Index, Wet Nm/g	Burst Index, kPa m^2^/g	Tensile Energy Absorption, J/m	Breaking Length, km	Stretch, %
KF	16.9	0.8	1.0	9.3	1.7	0.9
HF	60.4	10.9	4.6	25.2	6.1	3.4
FF	8.2	-	0.9	8.4	0.8	2.0
KF FF	13.9	1.3	1.0	17.7	1.4	2.0
HF FF	30.8	-	2.1	37.8	3.1	3.4
KF HF FF	35.9	1.9	2.8	54.3	3.7	3.9

**Table 4:** Results of elemental analysis of materials before and after composting test. KF––kraft fibers, HF––hemp fibers, and FF––fungus fibers.

Element, %	KF	HF	FF	KF FF	KF HF FF
C	42.5/41.9 *	41.7/43.7 *	38.9/45.5 *	40.5/43.2 *	40.9/41.9 *
H	5.9/5.8 *	5.8/5.9 *	5.8/5.5 *	5.8/5.7 *	5.8/5.7 *
N	0.2/0.2 *	0.3/0.9 *	1.8/2.7 *	0.9/1.5 *	0.7/1.8 *
C/N	189/175 *	126/50 *	22/17 *	45/29 *	62/24 *
ΔC/N **	8	60	24	36	62

* After the composting test. ** Difference in C/N ratio before and after composting test.

## Data Availability

The data presented in this study are available on request from the corresponding author.
